# Optimization of a new adaptive intervention using the SMART Design to increase COVID-19 testing among people at high risk in an urban community

**DOI:** 10.1186/s13063-022-06216-w

**Published:** 2022-04-14

**Authors:** Liliane Windsor, Ellen Benoit, Rogério M. Pinto, Jesus Sarol

**Affiliations:** 1 University of Illinois Urbana Champaign, Champaign, IL USA; 2 North Jersey Community Research Initiative, Newark, NJ USA; 3 University of Michigan, Ann Arbor, USA

**Keywords:** COVID-19 treatment cascade, Navigation services, Critical dialog, Brief counseling, Sequential Multiple Assignment Randomized Trials (SMART), COVID-19 testing

## Abstract

**Background:**

COVID-19 has impacted the health and social fabric of individuals and families living across the USA, and it has disproportionately affected people living in urban communities with co-morbidities, those working in high-risk settings, refusing or unable to adhere to CDC guidelines, and more. Social determinants of health (SDH), such as stigmatization, incarceration, and poverty, have been associated with increased exposure to COVID-19 and increased deaths. While vaccines and booster shots are available, it will take time to reach herd immunity, and it is unclear how long newly developed vaccines provide protection and how effective they are against emerging variants. Therefore, prevention methods recommended by the Centers for Disease and Control (CDC)—i.e., testing, hand-washing, social distancing, contact tracing, vaccination and booster shots, and quarantine—are essential to reduce the rates of COVID-19 in marginalized communities. This project will adapt and test evidence-based HIV interventions along the prevention and treatment cascade to help address COVID-19 prevention needs.

**Methods:**

The study aims to (1) optimize an adaptive intervention that will increase rates of testing and adherence to New Jersey State COVID-19 recommendations (testing, social distancing, quarantine, hospitalization, contact tracing, and acceptance of COVID-19 vaccination and booster shots) among high-risk populations and (2) identify predictors of testing completion and adherence to New Jersey recommendations. This study follows Community Based Participatory Research (CBPR) principles to conduct a Sequential, Multiple Assignment Randomized Trial (SMART) with 670 COVID-19 medically/socially vulnerable people. Participants will be recruited using a variety of strategies including advertisements on social media, posting fliers in public places, street outreach, facility-based, and snowball sampling. Participants complete a baseline survey and are randomized to receive navigation services or an electronic brochure. They then complete a follow-up 7 days after baseline and are randomized again to either continue with their original assignment or switch to the other intervention or critical dialog or brief counseling. Participants then complete a 5-week post-baseline follow-up. Guided by the COVID-19 Continuum of Prevention, Care, and Treatment, the analysis will explore the factors associated with COVID-19 testing within 7 days of the intervention.

**Discussion:**

This paper describes the protocol of the first study to use SMART following CBPR to adapt evidence-based HIV prevention interventions to COVID-19. The findings will inform the development of an effective and scalable adaptive intervention to increase COVID-19 testing and adherence to public health recommendations, including vaccination and booster shots, among a marginalized and difficult-to-engage population.

**Trial registration:**

ClinicalTrials.govNCT04757298. Registered on February 17, 2021.

## Administrative information

Note: The numbers in curly brackets in this protocol refer to SPIRIT checklist item numbers. The order of the items has been modified to group similar items (see http://www.equator-network.org/reporting-guidelines/spirit-2013-statement-defining-standard-protocol-items-for-clinical-trials/).

**Table:** 

Title {1}	Optimization of a new adaptive intervention using the SMART Design to increase COVID-19 testing among people at high risk in an urban community
Trial registration {2a and 2b}.	ClinicalTrials.gov, NCT04757298
Protocol version {3}	This manuscript reflects the original study protocol, version 1.0, which was accepted by the NJCRI IRB on February 16, 2021 (IRB Protocol number: UIUC-NJCRI 102497-18274-267805).
Funding {4}	3R01MD010629-04S2 (U.S. NIH Grant/Contract, National Institute on Minority Health and Health Disparities)
Author details {5a}	Liliane Windsor, Ph.D.University of Illinois Urbana Champaign Ellen Benoit, Ph.D.North Jersey Community Research Initiative Rogério M. Pinto, Ph.D.University of Michigan Jesus Sarol, Ph.D.University of Illinois Urbana Champaign
Name and contact information for the trial sponsor {5b}	National Institute of Minority Health and Health Disparities (Nathaniel Stinson: stinsonn@mail.nih.gov
Role of sponsor {5c}	The sponsor had no participation in the study design; collection, management, analysis, and interpretation of the data; writing of this manuscript; or decision to submit this manuscript for publication.

## Introduction

### Background and rationale {6a}

COVID-19 has impacted the health and social fabric of individuals and families living across the USA, and it has disproportionately affected people living in urban communities with co-morbidities, those working in high-risk settings, refusing or unable to adhere to CDC guidelines, and more [1, 2]. Social determinants of health (SDH), such as stigmatization, racial discrimination, xenophobia, incarceration, and poverty, have been associated with increased exposure to COVID-19 and increased deaths [3, 4]. Minoritized communities, defined as low-income and racial/ethnic minority neighborhoods, where residents experience increased barriers (e.g., inadequate housing, high-risk jobs) to prevention and treatment, bear disproportionately higher rates of co-morbidities associated with more severe cases of COVID-19 [3]. While effective and potent vaccines are becoming more available, it will take time to reach herd immunity, and it is unclear how long newly developed vaccines and boosters will provide protection and how effective they will be against emerging variants [5]. Therefore, prevention methods recommended by the Centers for Disease and Control (CDC)—i.e., testing, hand-washing, social distancing, contact tracing, vaccination, and quarantine—are essential to reduce the rates of COVID-19 in marginalized communities [6].

In the absence of evidence-based protocols to prevent and treat COVID-19, providers in primary care, community-based organizations, and other settings rushed to offer services while sheltering in place and keeping physical distance [7]. Such services, without much empirical evidence, did save many lives, but chaos in service settings was widely reported [8]. Uncoordinated approaches in vulnerable communities shed a harsh light on the need to improve integrated COVID-19 prevention services, particularly in Black and Latinx communities experiencing myriad health inequities, now exacerbated by COVID-19. Testing centers are more prevalent in White than in low-income predominantly Black neighborhoods. Black patients with COVID-19 symptoms are less likely than Whites with the same symptoms to receive a COVID-19 test [9–11]. The effects of COVID-19 on racial/ethnic minoritized communities include harms involving substance misuse, noncompliance with public health directives (hand-washing, shelter in place), suicidality, and unresolved grief from the loss of loved ones [12–14]. Meeting these multifaceted needs will require interventions that directly and effectively help individuals at-risk develop and maintain CDC-recommended COVID-19 prevention behaviors.

Research about COVID-19 testing and vaccine uptake in these communities must occur in real time, and it must account for the fast-changing landscape of the pandemic, including the impact of vaccine and booster availability on testing uptake. Two cost-effective, evidence-based, and culturally appropriate interventions have been effective in engaging people in HIV prevention and treatment—these can be adapted and tested to help address COVID-19 prevention needs. Specifically, Navigation Services (NS) have been shown to increase HIV testing and adherence to treatment while addressing structural barriers that deter treatment engagement in high-risk communities [15], and brief counseling (BC) has shown to increase HIV treatment engagement [16]. A third intervention, critical dialog (CD), showed promise in changing health risk behavior through critical thinking and group dialog [17].

#### Integrated framework and design overview

This study uses the well-known HIV Continuum of Prevention, Care, and Treatment Cascade (HIV CoPCT), a widely used framework in similar research, as a roadmap that guides clients from HIV testing to treatment to viral suppression [18]. Specifically, concerning prevention behaviors needed to accomplish the CoPCT, we are grounding this research in the capability–opportunity–motivation–behavior (COM-B) model [19]. The COM-B model assumes that behavior—social distancing, washing hands, inoculation, wearing masks, vaccination—occurs when both the capability and opportunity are present and when the person is motivated to enact that behavior. Combining COM-B and the CoPCT will allow us to assess the participants’ behavior change related to COVID-19 testing, immunization, and adherence to the CDC recommendations over time from testing and diagnosis to linkage to care through quarantine and recovery, with retesting when warranted. This study will further demonstrate the utility of the COM-B model and the CoPCT in tracking COVID-19 testing and follow-up in a medically and socially vulnerable population. Findings from our formative work, scientific literature, and experiential knowledge (drawn from long-term community research partners) informed our theoretical approach—i.e., working in groups to help individuals vulnerable to COVID-19 infection to obtain information about COVID-19 (brochure approach) or to access needed services (NS) and reinforcement via BC or CD. We will test which combinations of these strategies work best to help individuals get tested and adhere to prevention behaviors.

We ground our work in CBPR principles and methods in order to develop and test an adaptive intervention that will be assessed using a Sequential, Multiple Assignment, Randomized Trial (SMART) design [20–22]. CBPR is a paradigm for engaging community members in every step of the research process in order to bring direct benefits to those communities and enhance the quality of research [23]. This is accomplished through systematic knowledge-building activities within communities, such as trainings and critical group dialog, which seek to merge experiential and scientific knowledge. We also ground our work in the Multiphase Optimization Strategy (MOST), a pioneering framework for developing behavioral interventions that uses engineering principles to optimize efficiency, efficacy, and sustainability [21].

Adaptive interventions include a sequence in which the type or dosage of the intervention (e.g., number of NS sessions or whether to use a brochure or NS) is adjusted based on participant characteristics or responses (e.g., completing a COVID-19 test) [22]. Thus, an adaptive intervention allows treatment to be tailored to the specific needs of each participant. A SMART design involves multiple intervention stages, with each stage corresponding to one of the critical decisions involved in the adaptive intervention. In this study, the SMART design allows us to determine an empirically supported set of decisions on what interventions are most effective at different stages of the COVID-19 CoPCT. For instance, our adaptive intervention includes NS, information brochure, BC, and CD. These interventions address different COM-B. We are able to test if, for example, those participants experiencing structural barriers to COVID-19 testing may be best helped by NS while those who are hesitant to take the COVID-19 vaccine can be best helped by CD or BC.

### Objectives {7}

This study’s specific aims and hypotheses include the following:

1. Aim 1: To optimize an adaptive intervention that will increase rates of testing and adherence to COVID-19 CDC protocol (testing, social distancing, quarantine, hospitalization, and attitudes toward COVID-19 vaccination and booster shots) among high-risk populations, we hypothesize that:
  - Navigation Services (NS) will result in higher rates of COVID-19 testing compared to referral to testing.
  - For those who complete testing, NS will result in higher rates of adherence to CDC recommendations than to BC.
  - For those reluctant to get tested, CD will result in more people subsequently getting tested compared to the information brochure.
2. Aim 2: To identify predictors of testing completion and adherence to CDC recommendations.

### Trial design {8}

**Fig. 1:**
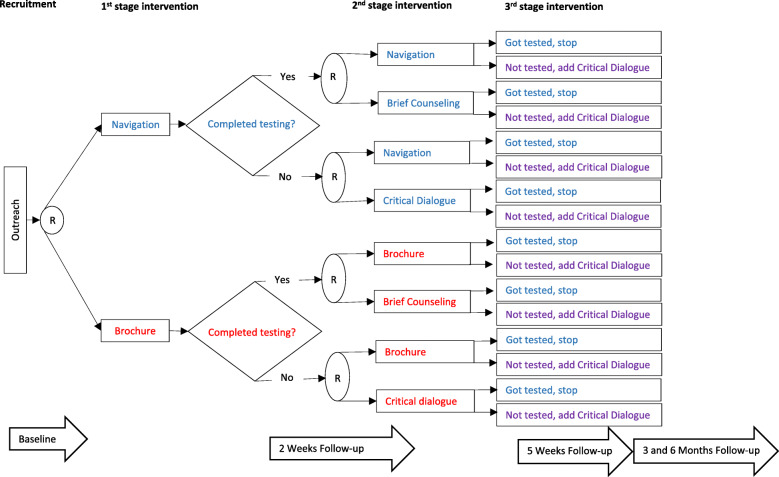
Trial design

This study follows CBPR principles and the MOST framework in implementing a SMART design with 582 COVID-19 medically and socially vulnerable people recruited in Essex County, NJ. The study’s approach builds on prior work using MOST [24] and CBPR [25] to develop and pilot test behavioral interventions with minoritized communities in Essex County, NJ. The study will be conducted by the Newark Community Collaborative Board, a group of service providers, consumers of health, community members, and researchers that have been working together since 2010 (NCCB; www.newarkccb.org). Guided by the HIV CoPCT, the analysis will identify the optimal combination of interventions for different participants and explore factors that promote or impede testing and adherence to CDC guidelines. Figure 1 illustrates the study design.

## Methods: participants, interventions, and outcomes

### Study setting {9}

The study will take place at the North Jersey Community Research Initiative (NJCRI, www.njcri.org). NJCRI is located in Newark, Essex County, NJ, one of the sites most heavily impacted by COVID-19 in the state of New Jersey. As of August 2020, Essex County had a rate of 2501 per 100,000 people testing positive for COVID-19 and 2111 deaths, the largest number in NJ. With a population of over 700,000, the county is 40% Black and 20% Latino. NJCRI is New Jersey’s largest and most comprehensive HIV/AIDS community-based organization, established in 1988 to provide clinical trials for investigational new HIV treatments. Populations served include youth and adults, men and women, men who have sex with men, people who acquire or who are at risk for HIV through injection drug use, and others. Other services include chronic illness management education, street outreach, substance abuse treatment, transportation, food pantry, and technical assistance to other organizations. NJCRI serves 10,000 people annually [26].

### Eligibility criteria {10}

The following are the inclusion criteria:

- Over 18 years of age
- Having a high risk to contract COVID or develop related complications
- Able to speak English
- Able and willing to provide informed consent

The following are the exclusion criteria:

- Under 18 years of age
- Not at a high risk to contract COVID or develop related complications
- Unable to speak English
- Unable or unwilling to provide informed consent

### Who will take informed consent? {26a}

Eligible individuals will provide written informed consent before participating in the study. During the informed consent process, the study outreach worker or research assistant will explain the study design in detail, including its purpose, the source of funding, why individuals are invited to participate, what they will be asked to do, how long the study will last, and how many people are expected to take part in the study. They will explain the processes of SARS antigen testing and randomization, the interventions being tested, potential sources of risk and discomfort, and measures designed to minimize both. They will answer all participant questions and will ask the participants to describe in their own words what they expect to do in the study before obtaining signatures. Participants who do not comprehend the study description will not be enrolled. Participants who would like some time to think before deciding will be able to take informed consent material home with them for review and consideration. All participants who provide written consent will receive a copy of the consent document which, in addition to the study description outlined above, includes names and contact information (phone numbers and email) for the principal investigators (PIs) and for the appropriate ethics officials at NJCRI.

### Additional consent provisions for collection and use of participant data and biological specimens {26b}

This is not applicable because this trial does not involve collecting biological specimens for storage.

### Interventions

#### Explanation for the choice of comparators {6b}

Factors that impact testing behaviors can be grouped into (1) structural factors (e.g., lack of transportation) and (2) individual factors (e.g., lack of information, personal beliefs) [27–31]. Thus, the first-stage interventions were selected to address these factors. Specifically, NS seek to address the structural barriers while also targeting the individual factors. A brochure only provides information. Hence, we will be able to determine if addressing both factors simultaneously is needed, or if for some participants, a brochure is sufficient. For participants that fail to get tested after exposure to first-stage interventions, we selected CD because it is designed to dispel myths and increase one’s ability to critically analyze information. We compare this intervention with BC because it is an evidence-based intervention for the delivery of results to those who get tested.

#### Intervention description {11a}

Participants are randomly assigned to NS or brochure after completion of the baseline. After the first follow-up survey, they are randomized again, either to continue the first intervention or to CD if they did not get tested or BC if they did get tested. Below, we describe each intervention.

##### Navigation Services

NS include assessment and support with service referrals. Each participant will meet with a peer navigator in person or on Zoom conferencing for 30 min to go over the results from their social and health needs assessment that is retrieved from the baseline survey. The navigator shares information about COVID-19, answers questions about testing, and makes referrals to other needed services. Follow-up sessions occur as needed.

##### Digital brochure

A digital brochure will consist of a brochure containing CDC public health recommendations that is e-mailed or texted to participants immediately after randomization. The brochure includes information about COVID-19, testing, and vaccines.

##### Critical dialog

CD includes three 1-h-long, open-group sessions facilitated in person or online by a trained licensed facilitator. Group critical dialog is prompted by thematic images developed by the NCCB to foster a deeper understanding of how systematic stigma, feelings of rage as victims of discrimination, and/or apathy may impact participants’ beliefs and behaviors related to COVID-19 and empower participants to make critical choices to protect their health and the health of their communities.

##### Brief counseling

BC is a 15-min post-COVID-19 test session delivered by a trained licensed clinician in person or via Zoom conferencing. In the session, the clinician shares the test results and offers recommendations and information about COVID-19 treatment and prevention.

##### Study arms

The study includes a total of eight arms as displayed in Table 1.

**Table 1:** Study arms

Study arm	Intervention 1	Tested for COVID-19 up to 7 days post-baseline	Intervention 2
1	NS	Yes	NS
2	NS	Yes	BC
3	Brochure	Yes	Brochure
4	Brochure	Yes	BC
5	NS	No	NS
6	NS	No	CD
7	Brochure	No	Brochure
8	Brochure	No	CD

#### Criteria for discontinuing or modifying allocated interventions {11b}

The prospect of testing positive for COVID-19 may be a cause of substantial fear and anxiety among participants. Many participants will have lost loved ones to COVID-19, and all of them will be medically or socially vulnerable to poor outcomes if they become infected themselves. It is also possible that participants who decline testing and attend CD sessions may be disturbed by discussions regarding stigma, racism, discrimination, and structural inequality. Differences among the group members have the potential to harm or cause discomfort for a participant or to revive psychological dynamics that could threaten to renew old conflicts and trauma. The peer navigators and licensed facilitators will be trained to address discomfort and trauma during the intervention sessions and participants are able to leave whenever they need. Clinical emergencies will be handled by the NJCRI treatment team of experienced staff. Appropriate assessments will be conducted; treatment options will be recommended and followed through as necessary. NJCRI is fully equipped to address any potential emergencies either themselves or via referrals. Any adverse event will be reported to the appropriate authorities within 48 h, and immediate action will be taken to minimize the likelihood of it occurring again. If a participant develops a serious illness and requires significant medical intervention and/or hospitalization, the study staff will do everything possible to ensure that the participant receives appropriate medical care. That participant’s follow-up interview(s) will be delayed until they are well enough to return to active participation. If a participant is unable to return or does not wish to return, they will be withdrawn from the study.

The NCCB will review preliminary findings presented by the PIs on a bi-monthly basis to address any potential adverse reactions that may occur and any safety concerns that may arise. If any participants are found to be unresponsive to the intervention, or getting worse during the course of the study, an NCCB subcommittee will discuss the case and the participant may be terminated from the study and referred to relevant services in the community. The PIs will meet weekly with study staff to review the study protocol adherence and address any potential problems. The PIs will also have weekly supervision meetings with intervention facilitators to review results from fidelity ratings and address any potential deviance from interventions.

#### Strategies to improve adherence to interventions {11c}

Participants will receive emails and texts from the study’s staff at regular intervals to remind them of appointments and encourage them to participate. Intervention adherence will be measured by the number of people who complete COVID-19 tests, number of sessions completed for each of the interventions, and receipt of the electronic brochure.

#### Relevant concomitant care permitted or prohibited during the trial {11d}

All participants will be able to seek care and interventions as they need during the study period.

#### Provisions for post-trial care {30}

There are no provisions for post-trial care. However, all participants will receive referrals to a variety of health and social services in the community.

### Outcomes {12}

Predictor and outcome standardized measures will be used to assess social determinants of health, demographics, COVID-19 risk (including comorbidities, exposure to SARS-CoV-2, adherence to prevention guidelines), attitudes about COVID-19 testing, health and social services used, knowledge about COVID-19, attitudes about COVID-19 vaccines and booster shots, mental health, protective factors and barriers to health care, confidence in government, and the healthcare system. Most of these measures will be selected from NIH PhenX Toolkit [32], and a portion of the measures is required by the NIH as part of the RADx-Up common data elements (CDEs) [33]. Qualitative in-depth interviews will focus on barriers to testing and attitudes about COVID-19 vaccination and recommendations for addressing those barriers.

Process measures include intervention fidelity, safety, quality assurance, and facilitator competence. Integrity assessments include (1) a checklist completed by facilitators at the end of each intervention session to list the activities discussed in each session and participant attendance and (2) measures of participants’ perceptions of helpful intervention components. The primary outcome is completion of the COVID-19 test within 1 week of the first intervention session. COVID-19 tests will be performed at NJCRI by a trained and licensed clinician. The date of test completion will be recorded in the study’s database. The final measure will consist of a dichotomous variable coded as 0 = no (people who did not get tested seven days post-baseline) and 1 = yes (people who got tested seven days post-baseline).

#### Participant timeline {13}

The participant timeline is presented in Table 2.

**Table 2:** Schedule of enrollment, interventions, and assessments

	***Recruitment***	***Baseline***	***Allocation1***	***Fu1***	***Allocation2***	***Fu2***
Time point**	***t***_***1***_	***t***_***2***_	***t***_***3***_	***t***_***4***_	***t***_***5***_	***t***_***6***_
**Enrollment**						
**Outreach**	**X**					
**Eligibility screen**	**X**					
**Informed consent**		**X**				
**Interventions**						
***NS***			**X**			
***Brochure***			**X**			
***BC***					**X**	
***CD***					**X**	
**Assessments**						
**COVID-19 knowledge (CDUHR)** [34]		**X**		**X**		**X**
**Attitudes about COVID-19 vaccine (CDEs)** [33]		**X**		**X**		**X**
**Attitudes about COVID-19 testing (CDEs)**		**X**		**X**		**X**
**Confidence in government and the healthcare system (CDUHR)**		**X**		**X**		**X**
**Comorbidities (CDEs)**		**X**		**X**		**X**
**HIV (CDEs)**		**X**		**X**		**X**
**COVID symptoms (CDEs)**		**X**		**X**		**X**
**Demographics (name, address, phone, email, social media, locator information, age, race, gender, sexual preference, marital status, income, education, disabilities)**		**X**		**X**		**X**
**Services accessed (TSR)** [35]		**X**		**X**		**X**
**Housing (TSR and CDEs)**		**X**		**X**		**X**
**Food access (PhenX Toolkit)** [32]		**X**		**X**		**X**
**Health care access (CDEs)**		**X**		**X**		**X**
**Use of technology (CDEs)**		**X**		**X**		**X**
**Social support (Mos Social Support abbreviated version** [36]**)**		**X**		**X**		**X**
**Pandemic impact (PhenX Toolkit)**		**X**		**X**		**X**
**Adherence to prevention guidelines (PhenX Toolkit)**		**X**		**X**		**X**
**Adherence to treatment guidelines (CDEs)**		**X**		**X**		**X**
**First COVID-19 test completion date**				**X**		
**Second COVID-19 test completion date**						**X**
**COVID-19 test 1 result; COVID-19 test 2 result**				**X**		
**General mental health (CDEs)**		**X**		**X**		**X**
**Violence and trauma (CDEs/PTSD Checklist 2005** [37]**)**		**X**		**X**		**X**
**Alcohol and nicotine use (CDEs)**		**X**		**X**		**X**
**Treatment fidelity**				**X**		**X**
**Client satisfaction**				**X**		**X**

#### Sample size {14}

For the primary aim of comparing effects of NS versus brochure on COVID-19 testing behavior, using standard sample size formula for the difference of two proportions, a total sample size of 582 subjects is needed to have 80% power at *α* = 0.05 to detect a 10% difference in proportions, assuming 70% in the brochure group will complete testing. The 582 sample size has 85% to detect a difference of 15% in testing rates between the two interventions. The study is not powered to detect changes in the second-stage interventions. Analysis of the second-stage interventions will estimate effect sizes and clinical significance of outcome change.

#### Recruitment {15}

We will enroll an intent-to-treat sample of 670 medically or socially vulnerable adults from urban Essex County, NJ. Adjusting for a possible 15% loss to follow-up yields a final sample of 582. The NCCB and staff outreach workers will post fliers at bus stops, health care agencies, churches, bulletin boards, and social service agencies. Individual service providers will disseminate information about the study. In addition, the research staff will encourage potential participants to help distribute the study’s fliers in their neighborhoods, religious institutions they attend, and other meeting places. Street outreach will follow the social distancing protocol. All NJCRI clients will be invited to screen, and those eligible will be invited to participate in the study. People interested in participating will call the study’s cell phone number. The staff outreach workers will conduct a brief phone screening to obtain self-reported eligibility information including measures of risk for contracting COVID-19 and developing related complications.

### Assignment of interventions: allocation

#### Sequence generation {16a}

The sequences of intervention assignments will be generated using PROC PLAN module in SAS Ver 9.4. Randomization of participants will be performed in two stages. In the first-stage, participants will be randomized to either the NS or brochure group with equal allocation. The assignment of participants to the intervention groups will follow a randomized permuted block design with a fixed block size of 8. In the second-stage, the participants will be stratified according to the intervention assignment and result of the first-stage intervention, forming six strata (NS tested positive, NS tested negative, NS not tested, brochure tested positive, brochure-tested negative, and brochure not tested). Within each stratum, participants will be randomized with equal allocation to one of two second-stage interventions which differed according to stratum. For NS tested positive or negative, the second-stage interventions are BC and continued NS. For NS not tested, the subjects are allocated to either continued NS or CD. For brochure tested positive or negative, the second-stage interventions are brochure and BC. For brochure not tested positive or negative, participants will be randomly allocated to either brochure or CD. Within each stratum, a randomized permuted block sequence of second-stage intervention assignments with a fixed block size of 8 will also be followed.

#### Concealment mechanism {16b}

The statistician will create a table of random numbers to be used by a research assistant. Once the participants complete the baseline, the research assistant will consult the table to allocate the participant to the intervention in REDCap.

#### Implementation {16c}

The outreach staff will recruit and enroll research participants and will have no role in the allocation of participants into interventions. The project statistician who is located at the University of Illinois and does not know the staff in New Jersey will generate the allocation sequence. A research assistant in Illinois will enter intervention assignments directly in REDCap.

### Assignment of interventions: blinding

#### Who will be blinded {17a}

Only data analysts will be blinded to the intervention assignments. They will receive a de-identified dataset that will list interventions with letters. They will not know what letters correspond to what interventions.

#### Procedure for unblinding if needed {17b}

Not applicable, only analysts will be blinded to the study conditions.

### Data collection and management

#### Plans for assessment and collection of outcomes {18a} Table 3

##### Intervention outcome measure

The primary outcome is dichotomous and operationalized as completing at least one COVID-19 test in the past month. The secondary outcome is adherence to the prevention guidelines in the past month operationalized with adherence questions from the Multi-Ethnic Study of Atherosclerosis (MESA) from NIH PhenX Toolkit [32].

##### Predictors

Standardized measures will be used to assess the social determinants of health, demographics, COVID-19 risk (including comorbidities, exposure to SARS-CoV-2), attitudes about COVID-19 testing, health and social services used, knowledge about COVID-19, attitudes about COVID-19 vaccines, mental health, protective factors and barriers to health care, confidence in government, and the healthcare system. Some of these measures were selected from the common data elements (CDEs) [33] required by the RADx-UP Coordination and Data Collection Center (CDCC), and others were selected from the NIH PhenX Toolkit [32].

##### COVID-19 testing capacity

FDA-approved nasal swab for SARS-CoV-2 antigen testing is currently offered at NJCRI and will be available free of charge for anyone enrolled in the study. NJCRI uses the LumiraDx SARS-CoV-2 antigen test which is authorized for use with nasal swab specimens and produces results within 15 min. NJCRI has the CLIA certificate and is able to conduct the laboratory processing in-house. NJCRI has been using LUMIRA over the past 1.5 years. Testing data will be obtained directly from NJCRI with written consent from participants (Table 3).

**Table 3:** Key study measures

Area of assessment	Variable measured	Measure source
Demographics	Name, address, phone, email, social media, locator info, Age, race, gender, sexual orientation, marital status, income, education, employment, housing, health insurance, language	CDEs [33]
COVID risk	Exposure to SARS-CoV-2	CDEs
	Comorbidities	
	Disabilities	
	COVID symptoms	
COVID-19 attitudes/beliefs/knowledge	COVID-19 knowledge	NIH PhenX Toolkit [32]
	Attitudes about COVID-19 vaccine	CDEs
	Attitudes about COVID-19 testing	
	Confidence in government and the healthcare system	PhenX Toolkit
Social determinants of health	Services accessed in the past 30 days/week/2 weeks	Treatment Service Review (TSR) [35]
	Housing	Added from TSR and CDEs
	Food access	PhenX Toolkit
	Health care access	CDEs
	Use of technology (including telehealth)	PhenX Toolkit/CDEs
	Social support	MOS Social Support [36]
	Pandemic impact	PhenX Toolkit
COVID-19 outcomes	Adherence to prevention guidelines	PhenX Toolkit
	Adherence to treatment guidelines	CDEs
	First COVID-19 test completion date	–
	Second COVID-19 test completion date	
	COVID-19 test 1 result	
	COVID-19 test 2 result	
	First stage intervention completion date	
	Second stage intervention completion date	
Mental health and drug use	Overall mental health	CDEs
	Trauma	CDEs and PTSD Checklist [37]
	Self-efficacy	General Self-Efficacy Scale (GSE-6) [38]
	Substance misuse	CDEs
Client satisfaction	End of treatment questionnaire	Developed by us
IRB	Anticipated adverse events	
	Serious adverse events	

Data will be collected on intervention fidelity, safety, quality assurance, and facilitator competence: Integrity measures assessments will include (1) a checklist completed by facilitators at the end of each intervention session to list the activities discussed in each session and participant attendance and (2) measures of participants’ perceptions of helpful intervention components.

#### Plans to promote participant retention and complete follow-up {18b}

To minimize attrition, participants will be asked to provide extensive locator information after each assessment, completing the form we used for the parent study, including formal and informal contacts who can reach the participant in a variety of contingencies. During the follow-up period, outreach workers will maintain weekly contact with participants by mail or phone and will update locator information.

#### Data management {19}

Data will be collected at the North Jersey Community Research Initiative. All data is stored in password-protected devices and/or locked in a filing cabinet in a locked office space where only authorized members of the research team have access. A database is set up using REDCap and Box Health in such a way that all authorized members of the research team will be able to access specific files remotely and securely through a HIPAA-compliant system. All team members received HIPAA training and were assigned a password-protected account that allows them to access the database. Dr. Benoit and Dr. Windsor are responsible for overseeing all data collection and processing. All identifying electronic data will be destroyed immediately after data collection is completed unless the participant agreed for us to retain the information so that they can have the opportunity to participate in future studies. In such cases, identifying information (name and contact information: e-mail, phone number, address, social media, alternative ways to contact) will be kept in a separate database in Box Health of potential human subject participants in future studies. Signed consent forms and audio files will be kept for 3 years after the study’s completion. De-identified electronic data will be kept indefinitely.

While this study does not seek to test the effectiveness of drugs or a medical device delivered in multiple sites, it will test an adaptive intervention in the community with a minoritized population and there is potential for harm. Thus, we developed a Data and Safety Monitoring Plan (DSMP) and the NCCB will serve as a Data Safety and Monitoring Board (DSMB) including clinical, intervention, community members, and statistical experts, to conduct periodic analyses of the evaluation data in the data collection phase.

Participants complete all surveys on a tablet or on their phones. The data is directly uploaded into REDCap. Prior to paying for study participation, the staff review the survey to ensure there are not unintentionally missed questions. The survey includes validation questions such as, “mark choice ‘c’ if you are reading this sentence.” The statistical team conducts a data analysis regularly to identify data entry mistakes. Any mistakes are sent back to the field staff for confirmation. Data collection error patterns are corrected as they are identified.

The NCCB will also conduct biannual monitoring of the data through spot checks and independent preliminary analysis of the data. For instance, the PIs and the project staff will meet regularly with project interviewers to review the assessments and coding of incoming data. The PIs will be responsible for ensuring that data are being coded, entered, and cleaned appropriately. The full research team will meet every week for 120 min to review all aspects of the research study.

#### Confidentiality {27}

To avoid possible violations of confidentiality, the project staff are trained to carefully follow detailed procedures designed to assure that no information about any participant (or others they may mention) will be given to anyone other than members of the research staff included in the IRB protocol. Every member of the research team and all NCCB members are required to undergo human subject protection training and provide a certificate of completion to the IRB prior to any contact with human participants. Standards of confidentiality include the use of code numbers and code names for participants and other individuals they may mention. The only place participants’ names or other identifying information will appear is on informed consent forms and locator forms that are password protected in REDCap, the software used in the study. This identifying information is used for follow-up interviews after the baseline, to remind participants of critical dialog group sessions if they are randomized to that condition and to locate participants if we are unable to reach them. Only the outreach staff, Dr. Benoit, and the group facilitator will have access to the identifying information.

The electronic files generated through data collection will be identified by code numbers only and kept in password-protected and HIPAA-compliant devices/cloud, separate from identifying information and available only to the project staff. This helps to ensure that no identifying information will be disclosed in the unlikely event that computerized data are stolen or otherwise seen by unauthorized persons. All identifying electronic data will be destroyed immediately after data collection is completed. Signed consent forms and audio recordings will be kept for 3 years after study completion. De-identified electronic data will be kept indefinitely. Study participants may ask at any time to have recordings of their interview destroyed by calling/e-mailing one of the PIs at the numbers provided in the informed consent forms.

Participants will be protected by a Certificate of Confidentiality issued by the NIH. Under terms of the Certificate, the researchers cannot be forced to disclose information that may identify a participant, even by a court subpoena, in any federal, state, or local civil, criminal, administrative, legislative, or other proceedings. However, the Certificate cannot be used to resist a demand from the United States Government personnel for information that is used for auditing or evaluation of federally funded projects or for information that must be disclosed in order to meet the requirements of the federal Food and Drug Administration (FDA). Researchers may also disclose the identity of participants who report intentions to harm themselves or others or if researchers have knowledge those participants are abusing children or elderly persons.

#### Plans for collection, laboratory evaluation, and storage of biological specimens for genetic or molecular analysis in this trial/future use {33}

Not applicable, we are not collecting biological specimens for this trial.

## Statistical methods

### Statistical methods for primary and secondary outcomes {20a}

Data analysis: Aim 1. We will estimate the (1) differences in the effects of first-stage interventions (NS vs brochure), (2) differences in the effects of second-stage interventions (BC vs NS among those who complete testing and referral vs CD among testing decliners), and (3) differences in the effects of selected adaptive interventions on acceptance of testing and adherence to NJ recommendations. For the differences between NS and brochure, cross-tabulations of the interventions with each outcome will be generated, and crude odds ratios comparing the two will be calculated. For the effects of second-stage and third-stage interventions, we will perform a similar analysis as that for the first-stage interventions but on corresponding subsets as indicated in the specific comparison groups. The estimation of effects of embedded adaptive interventions will use weighted and replicated estimation techniques [22, 39]. Under these procedures, the data set will be restructured wherein an individual’s outcome will be replicated in the data set depending on the number of adaptive interventions consistent with this outcome. Since randomization at both stages is based on equal probabilities, weighting is not necessary. The restructuring of the data set as described allows the utilization of standard statistical software for parameter estimation and hypothesis testing. A generalized linear model using logistic regression will be performed where estimation of model parameters will be done using generalized estimating equations [40] due to the presence of replicated subjects. We will then use tests of contrasts to compare selected adaptive interventions. The analysis of the adaptive interventions will be done using PROC GENMOD in SAS. To adjust for baseline characteristics and test for their moderating effects, we will incorporate these terms and their interactions with first-stage, second-stage, and adaptive interventions in their respective models. Only the baseline characteristics will be used for adjustment, as outcomes of the first-stage intervention are biased if incorporated into this model. Odds ratios, 95% CI, and Wald’s test for the effect of interventions and covariates will be obtained from these results. Sex/gender and race/ethnicity will be among these covariates that we will consider for adjustment and moderation analysis.

### Interim analyses {21b}

An interim analysis will be conducted bi-monthly by study analysts once data collection starts. This will include the development of syntax to clean the data (e.g., missingness analysis, identification of data entry errors, recoding variables). The key variables will be reviewed. Descriptive data will be presented to PIs and members of the NCCB. The NCCB will review preliminary findings presented by the PIs on a bi-monthly basis to address any potential adverse reactions that may occur and any safety concerns that may arise. If any participants are found to be unresponsive to the intervention, or getting worse during the course of the study, an NCCB subcommittee will discuss the case and the participant may be terminated from the study and referred to relevant services in the community. The PIs will meet weekly with the study staff to review the study protocol adherence and address any potential problems. The PIs will also have weekly supervision meetings with intervention facilitators to review the results from fidelity ratings and address any potential deviance from interventions.

### Methods for additional analyses (e.g., subgroup analyses) {20b}

Aim 2: To identify other predictors of these outcomes, we will include these variables as covariates using logistic regression analysis. We will model probabilities of testing behavior (completed vs testing decliners) and adherence to NJ recommendations as functions of the first-stage intervention and other covariates in the logistic regression analysis.

Qualitative component analysis: Critical dialog sessions and in-depth interviews will be digitally audio-recorded and transcribed. Data will be entered into Atlas.ti, a qualitative data processing program. Analysis rooted in phenomenology and grounded theory will include continuous coding, comparison, and recoding to yield categories and connect experiences and themes [41, 42]. The analysis will focus on participant reports about their experiences with the intervention, barriers to testing and adherence to NJ recommendations, and suggestions on how to address these barriers.

### Methods in analysis to handle protocol non-adherence and any statistical methods to handle missing data {20c}

We will perform an intent-to-treat analysis of the data. Subjects will be groups according to how they are randomized. The multiple imputation strategy described will be adapted [43]. This uses a time-ordered nested conditional imputation strategy with multiple imputation of missing values. In this approach, the fully conditional specification (FCS) imputation framework wherein a separate model for each variable is fitted will be applied. We will consider the time ordering of the data. For example, observed baseline variables will be used to impute for missing data from the first-stage results. Then, the imputed and observed values of the first-stage results will be used in conjunction with baseline variables to impute missing values for the second-stage results. We will examine the distribution of missing data according to intervention groups, covariates (e.g., demographic characteristics, adherence, reason for discontinuation), outcome variables, and time. We will determine whether the missing data pattern is not inconsistent with the missing-at-random (MAR) mechanism. We will also check whether the amount of missingness is monotonic over time. The r software packages *mice* and *pan* will be used for imputation [44, 45].

### Plans to give access to the full protocol, participant-level data, and statistical code {31c}

Consistent with the NIH policy, the data from this study will be made available, on appropriate terms and conditions, to the research community in a timely manner. We will follow the NIH policy on the Dissemination of NIH-Funded Clinical Trial Information NOT-OD-16-149. The study will be registered under the Clinical Trials Registration at ClinicalTrials.gov for public posting. The study will be registered within 21 calendar days after the enrolment of the first participant, results will be submitted no later than one year after the completion of final data collection, and all required elements will be provided. Results including participant flow, demographic and baseline characteristics, outcomes and statistical analyses, adverse events, the protocol and statistical analysis plan, and administrative information will be updated on ClinicalTrials.gov bi-annually (twice each study year). Informed consent documents for the study will include a statement providing information about the posting of clinical trial information on ClinicalTrials.gov.

Dr. Windsor will oversee the data storage and sharing procedures, and she will be the liaison with the RADx-UP Coordinating and Data Collection Center (CDCC). The University of Illinois at Champaign-Urbana will be the dedicated unit submitting reports on common evaluation metrics on COVID-19 testing-related outcomes and implementation to the CDCC. Data for this study will be collected through repeated quantitative surveys, observations, and intervention sessions compiled into REDCap and box. All databases will be stripped of personal identifiers, and code numbers will be replaced, to render data suitable for use by other investigators. Even though the database will be stripped of potentially identifying information prior to any sharing, we will make data and associated documentation available to users only through a controlled site and under a data-sharing agreement that includes a commitment to use the data only for research purposes and to protect privacy and confidentiality.

### Oversight and monitoring

#### Composition of the coordinating center and trial steering committee {5d}

This trial is being conducted by a consortium of organizations including the University of Illinois at Urbana-Champaign (grantee organization responsible for data management and analysis), the North Jersey Community Research Initiative (data collection site and IRB oversight), and the University of Michigan (home institution of the co-investigator responsible for overseeing the community engagement activities). Moreover, as part of the RADx-UP NIH initiative, the CDCC at Duke University provides support, consultation, and oversight of common data element data collection. Finally, the Newark Community Collaborative Board serves as the study’s DSMB.

#### Composition of the data monitoring committee, its role, and reporting structure {21a}

A DMC is not needed because this trial does not meet the criteria for the NIH phase II clinical trials. However, the NCCB is serving the study in the role of a DMC. A description of the NCCB can be found at: www.newarkccb.org. The organization is not independent from the principal investigators, but it is independent from the sponsors.

#### Adverse event reporting and harms {22}

Any research staff member who learns of an adverse event is responsible for reporting the event to the PIs(PI), who is in turn responsible for discussing the event to make a joint decision about whether the event is serious or non-serious. Events will be categorized as adverse or serious adverse according to NIH guidelines: “Adverse events (AEs) are defined as any untoward medical occurrence that may present itself during treatment or administration of an intervention, and which may or may not have a causal relationship with the treatment. Serious adverse events (SAEs) are defined as any medical occurrence that results in death; is life-threatening; requires inpatient hospitalization or prolongation of existing hospitalization; creates persistent or significant disability/incapacity, or a congenital anomaly/birth defects.” Adverse events (AEs) occurring during the course of the study will be collected, documented, and reported to the PIs. Each week a study investigator will review the AE Forms from the previous week for events that were reported as new or continuing. The study investigators will follow all AEs to the point of a satisfactory resolution. A study participant may be withdrawn from the study if the responsible investigator or group facilitators determine it is the best decision in order to protect the safety of a participant. All AEs will be assessed to determine if they meet the criteria for an SAE. If an AE meets the criteria for serious adverse event (SAE), as defined by the FDA, they will be systematically evaluated at each study visit and treated in a similar fashion as AEs with regards to monitoring and reporting.

Any SAE will be reported to the IRB within 48 h of the occurrence. The report of SAEs will include whether they were expected or unexpected, a rating of severity of the event, a brief narrative summary of the event, a determination of whether a causal relationship existed between the study procedures and the event, whether the informed consent should be changed as a result of the event, and whether all enrolled participants should be notified of the event. Finally, as part of the annual progress report (noncompeting continuation application) to NIH, we would provide summary information on all SAEs that have occurred during that year. Any actions taken by the IRB will be reported promptly to NIH via the project officer or appropriate parties.

#### Frequency and plans for auditing trial conduct {23}

PIs and project staff will meet weekly to review the recruitment data and discuss any concerns. The first author will meet with intervention facilitators bi-weekly to review intervention fidelity and challenging participant cases. The NCCB will meet bi-monthly to review reports of project implementation, including descriptive data.

#### Plans for communicating important protocol amendments to relevant parties (e.g., trial participants, ethical committees) {25}

Changes to the protocol will be submitted as amendments to the NJCRI IRB. Changes will only be implemented once the IRB amendment is approved. Changes will also be reported bi-monthly to the NCCB. Finally, common element data variables will be shared with the CDCC weekly.

#### Dissemination plans {31a}

The overall study results will be shared through publications in peer-reviewed journals and presentations at national scientific conferences (e.g., Society for Social Work Research, American Public Health Association). Study findings also will be shared with community-based organizations, through our community networks and via social media sites such as the NCCB’s website (https://newarkccb.org/).

UIUC also has an internal policy to ensure that all clinical trials registration and results reporting are in compliance with policy NOT-OD-16-149.

## Discussion

COVID-19 has impacted the health and social fabric of individuals and families living across the USA, and it has disproportionately affected people living in urban communities with co-morbidities, those working in high-risk settings, refusing or unable to adhere to CDC guidelines, and more [46]. Social determinants of health (SDH), such as stigma, racial discrimination, xenophobia, incarceration, and poverty have been associated with increased exposure to COVID-19 and increased deaths. Marginalized communities, defined as low-income and racial/ethnic minority neighborhoods, where residents experience increased barriers (e.g., inadequate housing, high-risk jobs) to prevention and treatment, bear disproportionately higher rates of co-morbidities associated with more severe cases of COVID-19 [47, 48]. While effective and potent vaccines and booster shots are becoming more available, it will take time to reach herd immunity and it is unclear how long newly developed vaccines provide protection and how effective they are against emerging variants. Therefore, prevention methods recommended by the CDC—i.e., testing, hand-washing, social distancing, contact tracing, vaccination, and quarantine—are essential to reduce the rates of COVID-19 in marginalized communities. Research about COVID-19 testing and vaccine uptake in these communities must occur in real time, and it must account for the fast-changing landscape of the pandemic, including the impact of vaccine availability on testing uptake.

Two cost-effective, evidence-based, and culturally appropriate interventions have been effective in engaging people in HIV prevention and treatment—these can be adapted and tested to help address COVID-19 prevention needs. Specifically, NS have shown to increase HIV testing and adherence to treatment while addressing structural barriers that deter treatment engagement in high-risk communities [15], and BC has shown to increase HIV treatment engagement [16].

This study uses a SMART with 582 COVID-19 medically/socially vulnerable people. Guided by the COVID-19 Continuum of Prevention, Care, and Treatment, the analysis will explore the factors associated with testing and adherence to public health recommendations. The study aims include the following: aim 1: to optimize an adaptive intervention that will increase rates of testing and adherence to NJ COVID-19 recommendations (testing, social distancing, quarantine, hospitalization, contact tracing, and acceptance of COVID-19 vaccination) among high-risk populations, and aim 2: to identify predictors of testing completion and adherence to CDC recommendations.

### Limitations

Due to methodological limitations (e.g., sample size), the examination of outcome changes beyond the first intervention stage will have low statistical power such that only big differences can be detected as statistically significant. Such findings will be interpreted with caution and these data will be used to estimate effect sizes for future studies. Moreover, reduction in health inequalities related to COVID-19 should follow increases in testing and adherence to CDC recommendations. Future studies will examine the impact of the optimized adaptive intervention on more outcomes. Future dissemination studies will have to test the intervention effectiveness in multiple settings.

In conclusion, this study is innovative in its application of existing evidence-based interventions to address COVID-19 and the use of SMART following community-based participatory research principles. It has public health relevance in that it will establish an effective and scalable intervention to increase COVID-19 testing and adherence to NJ recommendations among a marginalized and difficult-to-engage population.

## Trial status

Recruitment started on February 12, 2021, and it is expected to conclude at the end of February 2022.

## Abbreviations

AE: Adverse events
BC: Brief counseling
CBPR: Community Based Participatory Research
CDC: Centers for Disease and Control
CD: Critical dialog
CDCC: Coordination and Data Collection Center
CDEs: Common data elements
CDUHR: Center for Drug Use and HIV Research
COM-B: Capability–Opportunity–Motivation–Behavior
CoPCT: Continuum of Prevention, Care, and Treatment Cascade
COVID-19: Coronavirus disease 2019
FDA: Food and Drug Administration
HIV: Human immunodeficiency virus
IRB: Institutional Review Board
MOST: Multiphase Optimization Strategy
NCCB: Newark Community Collaborative Board
NJCRI: North Jersey Community Research Initiative
NIH: National Institutes of Health
NJ: New Jersey
NS: Navigation Services
PIs: Principal investigators
RADx-UP: Rapid Acceleration of Diagnostics-Vulnerable Populations
SAE: Serious adverse events
SDH: Social determinants of health
SMART: Sequential, Multiple Assignment Randomized Trial
TRS: Treatment Services Review
